# Scavenger Receptors: Novel Roles in the Pathogenesis of Liver Inflammation and Cancer

**DOI:** 10.1055/s-0041-1733876

**Published:** 2021-09-22

**Authors:** Daniel A. Patten, Alex L. Wilkinson, Ayla O'Keeffe, Shishir Shetty

**Affiliations:** 1 National Institute for Health Research Birmingham Liver Biomedical Research Unit, Institute of Immunology and Immunotherapy, University of Birmingham, Birmingham, United Kingdom; 2 Centre for Liver and Gastrointestinal Research, Institute of Immunology and Immunotherapy, University of Birmingham, Birmingham, United Kingdom

**Keywords:** scavenger receptors, macrophages, liver sinusoidal endothelial cells, cirrhosis, hepatocellular carcinoma

## Abstract

The scavenger receptor superfamily represents a highly diverse collection of evolutionarily-conserved receptors which are known to play key roles in host homeostasis, the most prominent of which is the clearance of unwanted endogenous macromolecules, such as oxidized low-density lipoproteins, from the systemic circulation. Members of this family have also been well characterized in their binding and internalization of a vast range of exogenous antigens and, consequently, are generally considered to be pattern recognition receptors, thus contributing to innate immunity. Several studies have implicated scavenger receptors in the pathophysiology of several inflammatory diseases, such as Alzheimer's and atherosclerosis. Hepatic resident cellular populations express a diverse complement of scavenger receptors in keeping with the liver's homeostatic functions, but there is gathering interest in the contribution of these receptors to hepatic inflammation and its complications. Here, we review the expression of scavenger receptors in the liver, their functionality in liver homeostasis, and their role in inflammatory liver disease and cancer.

## Liver Disease Pathophysiology

Liver disease is one of the leading causes of mortality in the United Kingdom, with cirrhosis becoming one of the top three causes of premature death in people aged 30 to 60 years. 1 Damage to the liver may originate from viral, metabolic, autoimmune, or toxin-induced pathways and etiologies differ depending on the site of injury. For example, nonalcoholic fatty liver disease (NAFLD)/nonalcoholic steatohepatitis (NASH) is initiated by lipotoxic damage to hepatocytes, 2 whereas primary sclerosing cholangitis arises from damage to cholangiocytes and leads to stricturing of bile ducts. 3 Nevertheless, regardless of etiology, most chronic adult liver diseases follow a common progressive pathophysiology in which persistent inflammation of the liver leads to the activation of hepatic stellate cells (HSCs), which in turn results in excessive production of extracellular matrix (ECM) proteins, leading to fibrosis, impaired liver function, and eventually cirrhosis. 4

Due to its increasing prevalence, chronic liver disease is fast becoming a serious global health burden. 5 6 In addition, chronic inflammatory disease significantly increases the risk of liver failure or hepatocellular carcinoma (HCC), with up to 90% of HCC cases arising on a background of cirrhosis. 7 Consequently, liver cancer is the seventh most common cancer worldwide and the fourth most common cause of cancer-related mortality. 8 Approximately three-quarters of all chronic liver diseases are diagnosed at a late stage when lifestyle interventions would be insufficient and there is also a distinct lack of effective treatments currently available for HCC; therefore, in both instances, transplantation is the only curative therapy. However, transplantation is clearly a major intervention with strict criteria and therefore is only considered a viable option in a minority of HCC patients. Those with advanced HCC have no curative options and despite the advent of immunotherapy, the majority of patients do not respond to current treatment. Consequently, there is an urgent need to better understand the inflammatory pathways and processes that contribute to liver disease progression so that novel targets may be identified and utilized.

Owing to its anatomical positioning in close association with the gastrointestinal tract, and frequently exposed to the vast milieu of gut-derived antigenic materials, the liver and its resident cell types express a wide array of scavenger receptors which are known to play key roles in homeostasis. In addition, there is now increasing evidence that scavenger receptors also play a diverse range of roles in inflammatory diseases of the liver and significantly contribute to their pathophysiology. In this review, we discuss the expression of scavenger receptors by various hepatic cells types, and their contribution to maintaining homeostasis and driving the pathogenesis of inflammatory liver disease and cancer.

## Scavenger Receptors

**Fig. 1:**
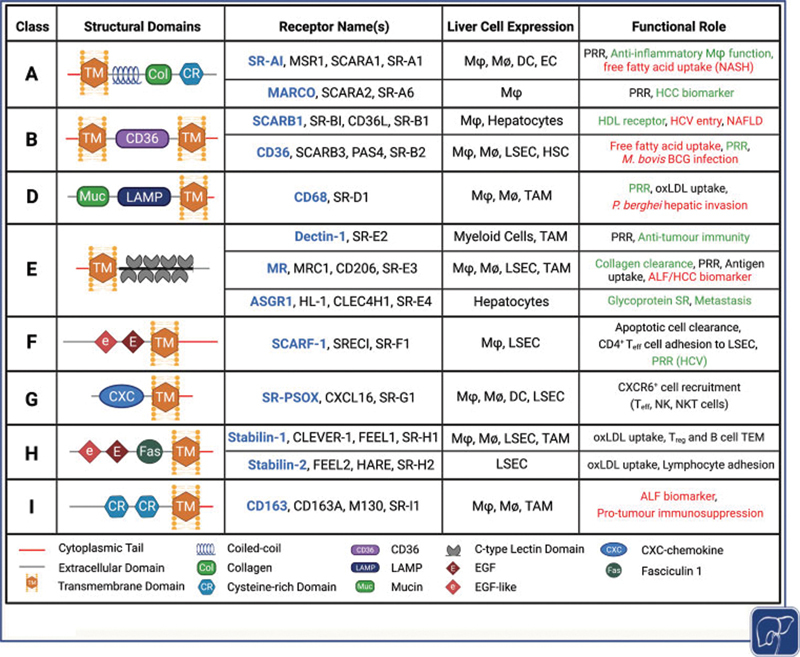
Scavenger receptors implicated in liver homeostasis and pathogenesis, including receptor class, shared structural domains, liver cell expression, and functional role within the liver. Functions shown in *green* are considered protective and those shown in *red* are considered detrimental. Context-dependent functions are indicated in *black* . Figure created using biorender.com. ALF, acute liver failure; DC, dendritic cells; EC, endothelial cell; EGF, epidermal growth factor; HCC hepatocellular carcinoma; HCV, hepatitis C virus; HDL, high-density lipoprotein; HSC, hepatic stellate cell; LAMP, lysosome-associated membrane glycoprotein; LSEC, liver sinusoidal endothelial cell; Mϕ, macrophage; M _Ø_ , monocyte; NK, natural killer cell; NKT, natural killer T cell; oxLDL, oxidized low-density lipoprotein; PRR, pattern recognition receptor; SR, scavenger receptor; TAM, tumor-associated macrophage; TEM, transendothelial migration; T _eff_ , T effector cell; T _reg_ , regulatory T cell.

Scavenger receptors are a diverse superfamily of evolutionarily-conserved receptors that play an important role in homeostatic processes, including nutrient exchange and waste clearance, and in immunity, such as inflammation regulation, leukocyte adhesion, and antigen presentation. 9 First identified by their ability to bind modified lipoproteins, such as oxidized low-density lipoproteins (oxLDLs), 10 scavenger receptors have now been shown to bind and/or internalize a diverse range of endogenous and exogenous ligands. 11 As such, scavenger receptors are now considered to be a subcategory of pattern recognition receptors (PRRs). 12 Scavenger receptors are categorized into classes A–J ( Fig. 1 ) depending on their structural and functional properties; however, there is little/no sequence homology between classes. 9

Although scavenger receptors are defined as cell-surface receptors, they may also be found intracellularly or as soluble forms in the circulation. 9 They can bind to a variety of unwanted self and non-self ligands and promote their removal from the systemic circulation via endocytosis, phagocytosis, or macropinocytosis. 9 11 Such ligands include damage-associated molecular patterns such as apoptotic cells, damaged proteins, cell debris, and heat shock proteins, and pathogen-associated molecular patterns, such as lipopolysaccharides (LPSs) and lipoteichoic acid. 13 Thus, their role in maintaining homeostasis spans beyond the scavenging of waste products, but rather, encompasses their ability to preserve appropriate levels of endogenous molecules while also recognizing foreign or damaging antigens and eliciting appropriate cellular immune responses.

The importance of scavenger receptors in homeostasis is highlighted by the complications that arise in their absence; for example, loss of functional SCARF1, SR-AI, or MARCO in transgenic mice leads to the development of systemic lupus erythematosus. 14 15 In addition, several scavenger receptors have been shown to play a key regulatory role in disease pathology, limiting the extent of injury in murine models of inflammatory disease. For instance, genetic deletion of SCARB1 was shown to increase disease severity in a murine model of Alzheimer's 16 and stabilin-1-deficient mice present with significantly more severe fibrosis in chronic liver injury models. 17 Conversely, some scavenger receptors have also been shown to drive the pathogenesis of multiple inflammatory diseases, such as atherosclerosis. 18 19

## Scavenger Receptors in Liver Homeostasis

### Scavenging of Endogenous and Exogenous Macromolecules

Scavenger receptors play a key role in maintaining homeostasis, particularly within the liver, which lies at the interface between the portal and systemic circulation. 20 Due to its anatomical positioning, the liver is constantly exposed to a myriad of gut-derived nutrients and microbial antigens and, as a result, it functions as the body's primary metabolic and detoxification system. As such, the liver-resident cells and their vast array of endocytic receptors, such as scavenger receptors, are integral for the removal of both endogenous and exogenous macromolecules and waste material from the circulation. The majority of this function is undertaken by liver sinusoidal endothelial cells (LSECs). 21

LSECs are highly specialized cells that line the liver sinusoids and represent the primary barrier between the bloodstream and underlying parenchymal tissues. Consequently, LSECs are adept scavengers, owing to their fenestrated morphology, lack of basement membrane, and superior endocytic capacity, and are involved in nutrient exchange, waste clearance, immune cell recruitment, and metabolism. 22 The primary scavenger receptors of LSEC are considered to be stabilin-1 and stabilin-2, which principally target oxLDLs for degradation, 23 amongst other macromolecules. Stabilin receptors (also known as FEEL/CLEVER/HARE) belong to the class H family of scavenger receptors, comprising a large extracellular N-terminus of 20 (stabilin-2) or 21 (stabilin-1) epidermal growth factor (EGF)/EGF-like domains, seven fasciclin-1 domains, and an X-linked domain, and a short intracellular C-terminal domain, linked by a transmembrane region 24 ( Fig. 1 ). Despite sharing 55% homology in their extracellular domains, these two receptors are distinguished by their highly diverse intracellular domains. 25 Although structurally distinct, both stabilin receptors mediate the specific hepatic uptake and clearance of oxLDLs by LSEC, an important mechanism for the prevention of atherogenesis. 26 Studies using fluorescent or radiolabeled oxLDLs demonstrated a predominant role for LSEC stabilin-1 in the uptake of circulating mildly oxidized LDLs, while stabilin-2 was suggested to recognize heavily oxidized LDLs, which are often found within atherosclerotic lesions. 23 Furthermore, mice deficient in stabilin-1 and stabilin-2 have a reduced lifespan and develop glomerular and hepatic fibrosis. 27 These studies highlight the homeostatic role for stabilin receptors, not just within the liver microenvironment, but at the systemic level.

LSECs have also been identified as the primary mechanism by which ECM components, such as collagen, are cleared from the circulation. Although both stabilin-1 and stabilin-2 have been implicated in the removal of profibrogenic circulating factors by LSEC, 27 the main receptor responsible for collagen clearance is thought to be mannose receptor (MR). 28 29 MR (also known as macrophage MR [MMR] and CD206) is a C-type lectin encoded by the MRC1 gene. Structurally, MR is composed of several domains within its large extracellular region, facilitating the binding of multiple ligands including lysosomal enzymes, 30 tissue plasminogen activator, 31 and mannose and *N* -acetylglucosamine residues commonly found on the surface of pathogens. 32 In mice, MR was shown to play a nonredundant role in the endocytosis of denatured collagen that occurred in a calcium-independent manner, which is consistent with a binding site distinct from that of mannose or mannan. 28 Furthermore, MR is important for maintenance of glycoprotein homeostasis, including clearance of advanced glycation end products. 33 This is exemplified in MR knockout mice which have impaired clearance and elevated circulating levels of mannosylated glycoproteins. 34

### Innate Immunity

In addition to the homeostatic clearance of unwanted endogenous ligands, scavenger receptors are also well characterized in their binding and internalization of a highly diverse range of exogenous antigens expressed by numerous bacteria, viruses, and fungi. 35 However, in spite of the extensive studies of the gut–liver axis as a key component of liver disease pathophysiology, 36 the role of scavenger receptors in the innate immunity of the liver remains largely unexplored.

Scavenger receptors on Kupffer cells have been implicated in the “fast clearance” of bacteria from the circulation 37 ; however, the specific scavenger receptor(s) involved remained undetermined by this study. Nevertheless, SR-AI in Kupffer cells has been shown to play a key role in the clearance of infection by the Gram-positive bacterium *Listeria monocytogenes* with SR-AI ^−/−^ mice exhibiting impaired uptake and killing of *L. monocytogenes* and larger, more frequent hepatic granulomas, compared with wild-type (WT) control mice. 38 CD36 in hepatic macrophages has been shown to play a role in the immune surveillance of nonbacterial pathogens, such as the fungal pathogen *Cryptococcus neoformans* . Significantly higher colony-forming units and significantly lower gene expression of inflammatory cytokines and chemokines were observed in livers of CD36 ^−/−^ mice compared with WT mice in a murine model of *C. neoformans* infection. 39 While several Kupffer cell-expressed scavenger receptors have been implicated in pattern recognition of several pathogens, they are not sufficient for effective systemic pathogen clearance, and so perhaps cannot be considered as phagocytic receptors per se. In contrast, complement receptor of the immunoglobulin family (CRIg), which is also highly expressed on Kupffer cells, is indispensable for the binding and clearance of complement component 3 (C3)-opsonized particles 40 41 and Gram-positive bacteria. 42 Furthermore, whereas some C-type lectins such as dectin-1 act as PRRs and endow the ability for phagocytosis, others, including MR, are not sufficient on their own to instill phagocytic capacity. 43 Moreover, despite many scavenger receptors possessing short intracellular domains that often lack a signaling motif, several act as components of signalosomes in complex with toll-like receptors (TLRs), integrins, and/or tetraspanins, including CD9 and CD81. 44 45 Indeed, scavenger receptors are known to form heterodimers with TLRs 44 46 47 and effectively boost the immune response to microbial antigens, compared with TLRs alone. 48 49 50 51 52 Thus, the role of scavenger receptors in innate immunity must be considered in the wider context of other innate immune and phagocytic receptors.

In contrast, some intracellular pathogens are able to hijack hepatic macrophage-expressed scavenger receptors to aid in their infection of the host. CD36 appears to play an active role in the pathogenesis of *Mycobacterium bovis* Bacillus Calmette-Guérin infection, with CD36 ^−/−^ mice exhibiting decreased mycobacterial burden and lower numbers of hepatic granulomas, and CD36 ^−/−^ macrophages restricting mycobacterial growth in vitro. 53 In addition, CD68 on Kupffer cells acts as a putative receptor for *Plasmodium berghei* sporozoites, 54 playing a significant role in the hepatic invasion stage of the malaria-causing parasite's lifecycle.

Hepatic macrophages have long been considered the major player in the hepatic innate immunity, 55 as they are largely responsible for the capture and clearance of pathogens from the bloodstream. 37 42 However, LSECs are known to play a key role in the clearance of bacterial LPS, 56 viruses, 57 and viral particles 58 from the bloodstream in vivo and have been shown to interact with several microbial antigens in vitro. 59 60 61 In fact, a recent study has implicated LSEC-expressed stabilin-1 and stabilin-2 in the systemic clearance of LPS 62 ; therefore, the role of LSEC-expressed scavenger receptors in hepatic innate immunity warrants future investigation.

### Immune Tolerance

LSECs are known to be highly efficient antigen presenting cells, 63 64 but rather than leading to T cell activation, they are skewed toward tolerance. 65 MR is a key player in promoting immune tolerance within the murine liver. Immune tolerance is critically important in maintaining hepatic homeostasis, due to the sustained exposure to low-level inflammatory agents from the gastrointestinal tract. For instance, uptake of oral antigens by MR has been shown to elicit tolerogenic responses following cross-presentation to CD8 ^+^ T cells. 63 66 Furthermore, antigen presentation by LSEC is known to induce CD8 ^+^ T cell tolerance via upregulation of co-inhibitory molecule programmed cell death ligand-1. 67 Notably, this has also been shown for tumor antigens, which led to tumor-specific CD8 ^+^ T cell tolerance. 68 69 This suggests that under physiological conditions, LSEC antigen presentation is important for maintaining liver tolerance, but that this could prove detrimental in a neoplastic context.

## Scavenger Receptors in Liver Disease Pathology

### Acute Injury

Acute liver failure (ALF) is the fulminant loss of liver function, which arises due to severe hepatic insult, usually in the absence of pre-existing liver disease. Often occurring due to exposure to noxious stimuli (e.g. drugs, alcohol, or viruses), ALF is characterized by jaundice, elevated circulating aminotransferases, and in severe cases, coagulopathy and hepatic encephalopathy. The most common cause of ALF is acetaminophen overdose which is thought to account for more than 50% of cases and approximately 20% of liver transplant cases. 70 71 Innate immune cell populations are key drivers of tissue damage in ALF, 72 yet despite their expression in these cell types and immunological functionalities being well documented, few studies have considered scavenger receptors in the context of ALF.

Some membranous scavenger receptors, such as CD163 and MR, are susceptible to proteolytic cleavage, leading to generation of soluble receptor forms which can retain some of their functional capacity. Often these are upregulated during inflammation and several have been characterized for their use as biomarkers of ALF. For instance, soluble CD163 (sCD163) is elevated in the circulation of ALF patients compared with healthy and cirrhotic patient controls, and is thought to represent increased intrahepatic macrophage activity. 73 74 75 In addition, higher and prolonged levels of sCD163 correlated with markers of hepatic dysfunction (bilirubin, creatinine, aspartate transaminase, alanine aminotransferase) and incidence of patient fatality. 74 Similarly, soluble MR (sMR) is increased in severe acetaminophen-induced liver injury. 75 A recent study has also reported elevated sCD163 and sMR levels in patients with acute-on-chronic liver failure, with both markers representing independent predictors of short- and long-term mortality. 76 77

To date, very few functional studies of the role of scavenger receptors in acute liver injury have been undertaken. However, studies of SR-AI showed its upregulation in myeloid cells in both human acute hepatitis and murine models of acute liver injury and subsequently demonstrated its protective role in this context. 78 79 Loss of SR-AI in transgenic mice was shown to significantly exacerbate liver damage in acute viral and hepatotoxin liver injury models, due to impairments in the anti-inflammatory activity of alternatively activated (“M2”) macrophages and myeloid-derived suppressor cells. 78 79

### Liver Inflammation

The extravasation of immune cells from the systemic circulation to the liver occurs within the hepatic microvasculature, known as the sinusoids, which are lined by highly specialized LSECs. Immune cell recruitment to vascular endothelial cells occurs via the leukocyte adhesion cascade, a complex and sequential multistep process involving numerous adhesion molecules and chemokines. However, in the low-shear flow environment within the liver sinusoids, this process is somewhat distinct from that which occurs within more conventional vasculature. In most other organs of the body, the selectins, a small family of transmembrane Ca ^2+^ -dependent lectins, play a major role in the initial rolling and adhesion steps of the leukocyte cascade. However, in the low-shear flow environment of the liver, the initial selectin-mediated rolling steps are not required and, consequently, their expression in LSEC is minimal. This absence of selectins allows for a greater contribution by more atypical adhesion molecules to the recruitment of leukocytes to the liver. In addition, the latter stages of the leukocyte adhesion cascade in which immune cells cross the endothelial barrier, known as transendothelial migration (TEM), differ considerably in the liver when compared with other organs. TEM is a multistep receptor-mediated process which requires strict regulation by the endothelial layer to prevent vascular leakage. TEM can occur via two highly distinct pathways: (1) the paracellular route in which leukocytes transmigrate via the cellular junctions between adjacent cells or (2) the transcellular route where leukocytes pass through the body of endothelial cells. Our previous studies with primary human LSEC have demonstrated that a significant proportion of lymphocytes migrate via the transcellular route, rather than the paracellular route as observed in more conventional endothelia. Several groups have now demonstrated that scavenger receptors can act as atypical adhesion molecules. Others and we have shown that LSEC-expressed atypical adhesion molecules, which are key in the recruitment of leukocytes to the liver in the diseased state, include certain scavenger receptors.

### LSEC-Expressed Scavenger Receptors and Leukocyte Recruitment

In addition to their primary scavenging function, several endothelial-expressed scavenger receptors have also been shown to exhibit a secondary functionality in which they act as atypical adhesion receptors in the leukocyte adhesion cascade. 80 The majority of these have been characterized in LSEC and implicated in the recruitment of leukocytes to the liver. 80

Perhaps the most well-studied of this subgroup of scavenger receptors is stabilin-1. 81 Stabilin-1 was first described as an atypical adhesion receptor in the trafficking of leukocytes across lymphatic vessels 82 83 and the same group later described its role in the TEM of lymphocytes through both lymphatic and vascular endothelial cells. 84 85 Our laboratory has previously characterized the expression of stabilin-1 in LSEC and explored its role in the TEM of lymphocytes to the liver. In our in vitro studies, which aim to mimic the physiological shear stress of the hepatic sinusoids, 86 we showed that stabilin-1 specifically mediated the TEM of both regulatory T cells (T _reg_ ) and B cells through primary human LSEC. 87 88 89 Despite stabilin-1 being implicated in the transmigration of specific immune cell subsets in a range of contexts, the leukocyte-expressed ligand(s) remain elusive.

Like stabilin-1, stabilin-2 has also been shown to be expressed in LSEC 90 91 92 and mediates lymphocyte recruitment to primary human LSEC in vitro. 91 However, despite significant sequence homology with stabilin-1, which as discussed above plays a role in the transmigration step of lymphocyte recruitment to LSEC, it has been suggested that stabilin-2 acts in the earlier stages of the leukocyte adhesion cascade, mediating the firm adhesion of lymphocytes. 91 In addition, and again in contrast to stabilin-1, a lymphocyte-expressed ligand to stabilin-2, α _M_ β _2_ integrin, has been identified. 91 Surprisingly, only one study to date has demonstrated the adhesive function of stabilin-2 and, given that other immune cell subsets, such as monocytes and neutrophils, also express α _M_ β _2_ , future studies could aim to elucidate whether or not stabilin-2 also mediates the recruitment of myeloid cells to LSEC.

Scavenger receptor that binds phosphatidylserine and oxidized lipids (SR-PSOX) is the membrane-bound form of chemokine CXCL16 and, consequently, binds CXCR6 ^+^ leukocytes in a highly specific manner. 93 94 95 96 SR-PSOX is thought to support leukocyte adhesion by triggering the conformational activation of immune-cell-expressed β1 integrins and acts in the “arrest” stage of the leukocyte adhesion cascade. 97 In the context of hepatic inflammation, SR-PSOX has been shown to interact with several proinflammatory intrahepatic immune cell subsets, such as effector T cells, 97 98 natural killer (NK) cells 99 100 and NK T cells, 101 all of which express CXCR6. In murine models of acute liver injury, pharmacological targeting of SR-PSOX with neutralizing antibodies and genetic deletion have been shown to significantly attenuate the intrahepatic inflammation and level of injury, 102 103 104 thus highlighting its therapeutic potential. 105

Another endothelial-expressed scavenger receptor which has previously been described in murine LSEC 106 and, more recently, in human LSEC 60 is SCARF1. By utilizing physiological flow-based adhesion assays with immobilized recombinant SCARF1 and antibody-inhibited or siRNA-silenced primary human LSEC, SCARF-1 was shown to mediate the adhesion of CD4 ^+^ T cells to LSEC. 60 In addition, the role of SCARF1 was CD4 ^+^ subset-specific with preferential binding to proinflammatory “effector” CD4 ^+^ T cells, rather than their regulatory CD4 ^+^ CD25 ^+^ counterparts (T _reg_ ). 107 These studies ruled out the possibility of a homophilic interaction, as CD4 ^+^ T cells did not express SCARF1, but the ligand is yet to be identified.

The role of scavenger receptors as atypical adhesion molecules is interesting to consider in the context of recruitment via conventional adhesion molecules such as intercellular adhesion molecule 1 (ICAM-1) and vascular cell adhesion molecule 1 (VCAM-1). Conventional adhesion molecules have a significant role to play in regulating the immune microenvironment in liver diseases. 108 109 Recent work has confirmed this in models of fatty liver disease, where VCAM-1 and ICAM-1 expressed on LSEC have been implicated in regulating proinflammatory monocyte recruitment. 110 It is therefore possible that scavenger receptors may amplify or modify the roles of conventional adhesion molecules. For example, in human in vitro models, T _reg_ TEM was inhibited by 50% by ICAM-1 blockade but with the combination of stabilin-1 blockade this was increased to 80% inhibition. 87

### Viral Hepatitis

Viral hepatitis is a global health problem, accounting for over a million deaths worldwide each year, 111 with a large proportion of those deaths attributed to hepatitis C virus (HCV). 112 Hepatic inflammation resulting from HCV can manifest from acute or chronic infection and is a major cause of liver cirrhosis and HCC. 113 HCV is a hepatotropic RNA virus and direct infection of hepatocytes is largely responsible for the resultant inflammatory disease. 114 115 116 While patients with HCV can now undergo highly effective therapy to eradicate the virus in symptomatic disease, there is still no preventative vaccine available and often infection leads to asymptomatic liver disease that can present at advanced stages with cirrhosis and HCC.

In normal physiology, scavenger receptor class B member 1 (SCARB1) on hepatocytes acts as receptor for high-density lipoproteins and plays a key role in cholesterol homeostasis. 117 However, in the context of viral hepatitis, SCARB1 is one of four receptors known to act as a HCV entry factor in human hepatocytes 118 119 120 121 and binds to HCV via its envelope glycoprotein E2. 122 Consequently, SCARB1 is strongly implicated in the pathogenesis of viral hepatitis and this is emphasized by the fact that patients with genetic variants of SCARB1 exhibit altered viral load 123 and virological responses. 124 125 Nevertheless, therapeutic targeting of SCARB1 with an antagonist in early-phase clinical trials demonstrated relatively low efficacy in HCV patients, suggesting a certain level of redundancy in its role. 126 127 In addition, and consistent with scavenger receptors being considered PRRs, one study has implicated both SR-AI and SCARF1 in the uptake and cross-presentation of HCV nonstructural (NS)3 protein by human myeloid cells (dendritic cells [DCs] and monocytes). 49

### Fibrosis

Hepatic fibrosis is the process in which the excessive accumulation of ECM proteins, such as collagens, fibronectin, and elastin, effectively replaces the parenchymal tissues of the liver, thus significantly distorting its histology and vastly reducing its functionality. 128 The major source of these ECM proteins is the HSC, a liver-resident pericyte which, when chronically activated by paracrine inflammatory signals from the diseased liver microenvironment, transdifferentiates toward a profibrogenic myofibroblast phenotype. 129 It has previously been suggested that direct HSC uptake of oxLDLs via CD36 and LOX-1 contributes to their activation and production of ECM proteins. 130 131 In addition, one study has suggested that Class A scavenger receptors may play a role in HSC activation in response to apoptotic bodies derived from HCV-infected hepatocytes. 132

Stabilin-1 expression is absent from HSCs; however, its presence/absence on other hepatic cell types is still able to indirectly influence HSC activation and hepatic fibrosis in vivo. Stabilin-1-deficient mice are phenotypically normal and exhibit a comparable lifespan to WT littermates 27 ; however, histological analyses of their livers demonstrated the presence of a mild, peri-sinusoidal deposition of collagen fibers. 17 27 This was indicative of a role for stabilin-1 in hepatic fibrogenesis and subsequent studies in the context of chronic liver injury further highlighted this. Utilizing a chronic carbon tetrachloride (CCl _4_ ) model of bridging hepatic fibrosis with a resolution phase, these studies demonstrated that a lack of stabilin-1 exacerbated the level of fibrosis and delayed its resolution. 17 In uninjured liver tissues, stabilin-1 expression is limited to endothelial cells 87 ; however, in the context of inflammatory disease, a sub-population of stabilin-1 ^+^ macrophages was also evident. 17 Cell-specific knockout mice (ENDO stab-1 ^−/−^ and MACRO stab-1 ^−/−^ ) were used to confirm that stabilin-1 in macrophages was the key contributor to the limitation of fibrosis in chronic injury. Additionally, adoptive transfer of stabilin-1-expressing myeloid cells, derived from bone marrow of WT mice, into MACRO stab-1 ^−/−^ mice was able to rescue the phenotype. Mechanistically, the scavenging of oxLDLs, and more specifically malondialdehyde (MDA)-LDLs, by macrophage-expressed stabilin-1 resulted in the suppression of CCL3 production. CCL3 is a proinflammatory and profibrotic chemokine known to influence fibroblast phenotype and its increased expression from macrophages in the livers of stab-1 ^−/−^ and MACRO stab-1 ^−/−^ mice led to increased fibrosis and delayed resolution. 17

### Steatosis and NASH

NAFLD is characterized by the chronic and excessive accumulation of lipids within the liver and is the precursor to a progressive and inflammatory form of the disease, NASH. 133 Given the global obesity crisis, NASH is increasing in prevalence and causes an incredible disease burden and economic impact worldwide. 133 NASH is classically characterized by steatosis, inflammation, and fibrosis, and a recent study has implicated SR-AI in the progression of NAFLD to NASH. 134 Govaere et al showed that SR-AI expression strongly correlated with the degree of steatosis and inflammation in a large cohort of NAFLD patients and, by utilizing a combination of murine models, novel ex vivo human liver tissue models, and in vitro experiments, they demonstrated a direct role for hepatic macrophage-expressed SR-AI in the uptake of free fatty acids, subsequently driving them toward a proinflammatory phenotype. 134 SCARB1 has also been demonstrated to be significantly upregulated in both murine and human NAFLD 135 and mice deficient in SCARB1 were recently shown to have significantly reduced levels of hepatic triglycerides in comparison to WT mice in a murine model of NAFLD. 136

However, the best studied scavenger receptor in context of lipid metabolism, steatosis, and NAFLD/NASH is CD36. 137 CD36 is widely expressed in the liver and has been studied in a range of hepatic cell types, including hepatocytes, 138 139 HSC, 130 LSEC, 140 Kupffer cells, 141 142 and even intrahepatic lymphocytes. 143 CD36 has consistently been shown to be significantly upregulated in NAFLD and NASH liver tissues in comparison to normal liver control tissues 144 145 146 ; however, the full significance of its role in NAFLD/NASH pathogenesis is still to be realized. 137 Nevertheless, overexpression of CD36 in murine livers significantly increased accumulation of hepatic triglycerides and cholesteryl esters in a model of diet-induced obesity 147 and hepatocyte-specific deletion of CD36 protected mice from steatosis and improved insulin sensitivity in a high-fat diet liver injury model. 148 In addition, the soluble form of CD36 may present as a biomarker for steatosis in NAFLD. 149 150

## Scavenger Receptors in HCC

### Tumor Endothelial Cell Expression of Scavenger Receptors

Several scavenger receptors have previously been implicated in the pathogenesis of a range of cancers 13 and HCC is no exception to this. In general, scavenger receptors are considered to be largely anti-inflammatory and, therefore, potentially pro-tumorigenic in nature; however, a seminal study demonstrated a key role for SR-PSOX/CXCL16 in LSEC-mediated recruitment of antitumoral NKT cells. 151 The results of this study showed that disruption of the commensal gut microbiota and accumulation of primary bile acids in the liver upregulated the expression of SR-PSOX/CXCL16 on LSEC, mediating the recruitment of CXCR6 ^+^ NKT cells which in turn effectively limited tumor formation and metastasis in mice. 151 More recently, studies on the expression of SCARF1 in tumor endothelial cells in human HCC tissues demonstrated a correlation with lower tumor aggressiveness, better survival, and increased inflammation. 107 From a functional perspective, the data suggested that SCARF1 potentially plays a role in the selective recruitment of proinflammatory “effector” CD4 ^+^ T cells which could initiate an antitumoral immune response. 107

Stabilin-1, which has been shown to recruit T _reg_ , is also expressed in tumor endothelial cells in human HCC tissues. 87 This suggests that the presence of stabilin-1 in HCC could be immunosuppressive and protumorigenic, a hypothesis which is supported by murine tumor models. In these studies, the genetic and therapeutic targeting (via antibody blockade) of stabilin-1 resulted in smaller primary and metastatic tumors and diminished numbers of immunosuppressive leukocytes, such as T _reg_ . 152

### Scavenger Receptors in TAMs

In addition to transformed cancer cells, the tumor microenvironment (TME) in HCC is composed of several other stromal components, including immunosuppressive tumor-associated macrophages (TAMs) which drive tumor growth, survival, and metastasis. 153 154 These TAMs are highly plastic and thought to originate from circulating monocytes, whose phenotype is shaped by the environmental cues found within the TME. The “M2” or “alternative activation” state of TAMs is well documented, and has been shown to promote an immunosuppressive phenotype, which can facilitate tumor immune evasion in HCC. 155 156

MR is considered a definitive marker of the “M2” TAM population 157 and has, unsurprisingly, been shown to be expressed in TAMs within HCC tumor tissues, with their presence being highly indicative of poor patient prognosis. 158 159 However, the direct contribution of MR to the TME in the context of HCC remains unexplored to date. Nevertheless, MR is known to directly bind tumoral mucins and this is speculated to drive immunosuppression by interleukin (IL)-10-mediated T _reg_ induction and downregulation of IL-12. 160 161 Furthermore, targeting of TAM-expressed MR with a synthetic peptide analogue in murine xenograft models resulted in M2 macrophage reprogramming to an antitumor “M1” phenotype, as well as inducing apoptosis of the M2 TAM population. 162 These studies demonstrate the potential for targeting TAM-expressed MR in the context of HCC and future studies should explore this possibility.

Similar to MR, CD163 is also a marker of immunosuppressive TAMs, which has been demonstrated in multiple cancers. 163 164 165 166 CD163 is the prototypic member of the class I family of scavenger receptors which bind and clear hemoglobin–haptoglobin complexes. 167 168 169 As such, this receptor prevents hemoglobin-induced inflammation under physiological conditions, but can also stimulate protumorigenic M2 polarization in a pathophysiological context. Specifically, hemoglobin release into the circulation, arising from pathological intravascular hemolysis within the tumor, can activate stress-responsive enzyme heme oxygenase 1. 169 This enzyme has been implicated in M2 polarization and IL-10 production. 170 171 Moreover, CD163 is involved in the sequestration and subsequent inactivation of proinflammatory tumor necrosis factor-like weak inducer of apoptosis (TWEAK), further contributing to the TAM immunosuppressive phenotype. 172 In support of this, enhanced peritumoral CD163 has been shown to correlate with poor prognosis and higher incidence of vascular invasion in HCC patients. 154 173

Another scavenger receptor highly expressed in TAMs is stabilin-1. 174 TAM stabilin-1 has been shown to scavenge antitumor factor, secreted protein, acidic and rich in cysteine (SPARC), which promoted tumor progression in an in vivo model of breast cancer. 174 Studies in mice have highlighted that genetic stabilin-1 deficiency leads to reduced intratumoral “M2” macrophages and FoxP3 ^+^ T _reg_ , demonstrating a role for stabilin-1 in shaping the immunosuppressive TME. 152 Importantly, macrophage-specific deletion of the stabilin-1 gene reduces tumor growth and metastatic spread, and anti-stabilin-1 antibody treatment of WT mice inhibits tumor progression. 152 Furthermore, stabilin-1 levels positively correlate with resistance to immune checkpoint therapies and T cell dysfunction in numerous cancer types. 175 Recently, a phase I/II first-in-man clinical trial in advanced solid tumor cancer patients including HCC demonstrated that the targeting of stabilin-1 with a function blocking antibody led to a significant phenotype switch in circulating monocytes. Interestingly, early results in patients where biopsies were undertaken did show a reduction of anti-inflammatory intratumoral stabilin-1 ^+^ macrophages and an increase in proinflammatory/adaptive immune cell subsets in selected patients. 176 This trial is ongoing and overall therapeutic efficacy is awaited, yet the data are consistent with the detrimental effects of TAM scavenger receptors and their role in driving an immunosuppressive TME, and also highlight therapeutic potential for their targeting within the context of HCC.

Conversely, scavenger receptors found on TAMs can also drive protective antitumor responses. For instance, dectin-1, a C-type lectin belonging to the class E family of scavenger receptors, can stimulate both innate and adaptive arms of the immune system to enhance immune-mediated tumor cell killing. Dectin-1 is the primary β-glucan receptor on myeloid DCs, macrophages, monocytes, and B cells and is upregulated in both DCs and TAMs within murine HCC tumors, when compared with normal liver tissues. 177 In addition, treatment of TAMs with β-glucan has been shown to convert “M2” polarized macrophages into an “M1-like” phenotype in a dectin-1-dependent manner. 178 Moreover, oral β-glucan treatment enhanced effector T cell activation and delayed tumor growth in mice. 178 Furthermore, recognition of tumor cells by DC- and macrophage-derived dectin-1 drives tumoricidal activity of NK cells and induces inflammatory cytokine production. 179 180 Consistent with this, dectin-1-deficient mice display exacerbated tumor growth in a murine model of HCC. 177 Thus, TAM scavenger receptors have a pleiotropic role in mediating the TME.

### Scavenger Receptors in Metastasis of HCC

The majority of scavenger receptors within the context of HCC are expressed in tumor-associated stromal and immune cell populations, such as tumor endothelia and TAMs; however, one scavenger receptor known to be expressed directly by tumor cells in HCC is asialoglycoprotein receptor 1 (ASGR1). 181 182 ASGR1 is a hepatic C-type lectin receptor which is constitutively expressed in hepatocytes and its primary function is to mediate the endocytosis of serum glycoproteins, particularly those containing galactose or *N* -acetylgalactosamine moieties. 183 184 ASGR1 expression was found to be downregulated in HCC tumors when compared with matched nontumorous tissues; however, higher intratumoral expression of ASGR1 in HCC was shown to be associated with better patient survival. 185 The same study also demonstrated that ASGR1 works in conjunction with longevity assurance homolog 2 of yeast LAG1 (LASS2) to inhibit vacuolar H ^+^ -ATPase (V-ATPase) activity in HCC tumor cells, effectively suppressing cell migration and invasion (i.e., metastasis).

Similarly, the intratumoral expression of another scavenger receptor, MARCO, has been shown to be associated with better patient prognosis 186 and its overexpression in hepatoma cell lines appeared to inhibit their migration and invasive properties, inducing apoptosis both in vitro and in vivo. 186 However, unlike ASGR1, which seems to be exclusively expressed on hepatocytes and HCC tumor cells, the expression of MARCO is also well known to be largely expressed by hepatic macrophages 187 188 ; therefore, more in-depth studies are required.

### Clinical Translation and Future Perspectives

**Fig. 2:**
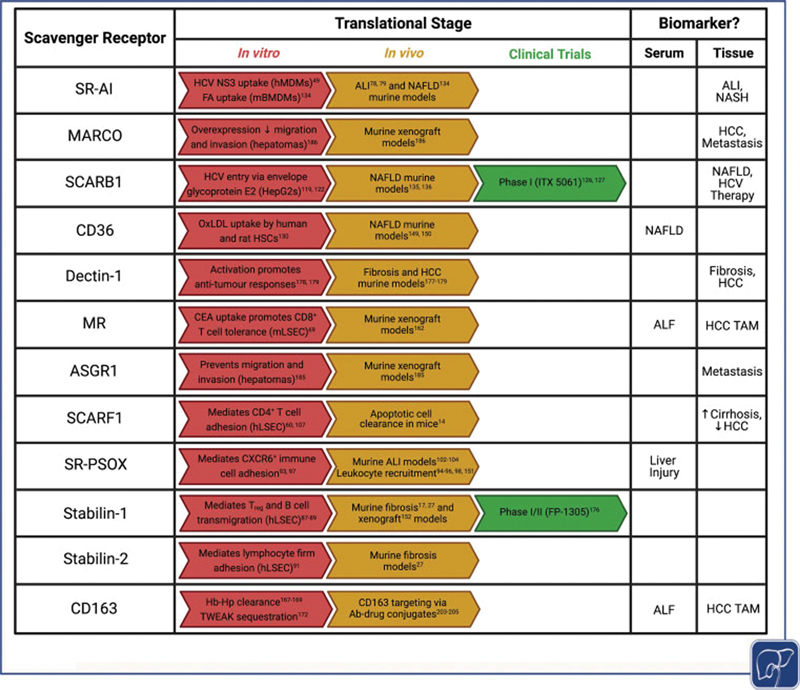
Scavenger receptors represent valid therapeutic targets and are often valuable for use as biomarkers of liver disease, either in their soluble form in the serum or their cellular form within the tissues. This table summarizes the translational stage of each scavenger receptor, including in vitro and in vivo preclinical models and ongoing or completed clinical trials. Figure created using biorender.com. ALF, acute liver failure; ALI, acute liver injury; CEA, carcinoembryonic antigen; FA, fatty acid; Hb-Hp, hemoglobin–haptoglobin complex; HCC, hepatocellular carcinoma; HCV, hepatitis C virus; hLSEC, human liver sinusoidal endothelial cells; hMDMs, human monocyte-derived macrophages; HSC, hepatic stellate cells; mBMDMs, murine bone marrow-derived macrophages; mLSEC, murine liver sinusoidal endothelial cells; NAFLD, nonalcoholic fatty liver disease; NASH, nonalcoholic steatohepatitis; NS3, nonstructural 3 protein; oxLDL, oxidized low-density lipoprotein; TAM, tumor-associated macrophage; T _reg_ , regulatory T cells; TWEAK, tumor necrosis factor-like weak inducer of apoptosis.

Over the last few years, scavenger receptors have increasingly been considered viable clinical targets 80 189 190 191 ; however, their full translational potential is yet to be realized as clinical trials have been limited in this field. In Fig. 2 , we summarize the translational progress in the study of scavenger receptors in liver disease, with two early-phase clinical trials active/completed to date. The first of these targeted scavenger receptor SCARB-1 in HCV cell entry in the context of liver transplantation 127 and the second is an early-phase clinical trial currently underway, targeting the scavenger receptor stabilin-1 on TAMs in solid tumors, including HCC. 176 In addition, scavenger receptors have also been explored as biomarkers of disease states, and here we highlight their potential as biomarkers in different etiologies of liver disease ( Fig. 2 ).

Nevertheless, there are still several questions that need to be explored before we can fully understand the contribution of scavenger receptors to liver disease and neoplasia, their therapeutic potential, and their practicality as disease biomarkers. While scavenger receptors share overlapping ligand recognition, the cellular responses can be divergent. 9 Better understanding is therefore needed of the cell signaling that takes place when specific scavenger receptors bind to their ligands to design therapies/small-molecule inhibitors that can take advantage of scavenger receptor biology. Detailed understanding is also needed of receptor dynamics, shedding, and recycling in homeostasis and how this may differ in disease settings, which will provide crucial information for pharmacokinetics and pharmacodynamics of drug design. With regard to liver disease, targeting of the complex tissue microenvironment remains a challenge in the setting of chronic hepatitis and neoplasia. Further studies are required to understand the contributions of scavenger receptors to persistent inflammation which drives liver fibrosis, and in contrast, the suppression of hepatic immune responses that permit the growth of tumors in the case of HCC and cholangiocarcinoma. One emerging technology which could aid in this process is single-cell RNA-sequencing (scRNA-seq). Recent scRNA-seq studies have highlighted tissue-resident and infiltrating immune cell populations, as well as inflammation- and cancer-associated hepatic cell subsets in human and mouse liver tissues. 192 193 194 Data generated from this technology could be utilized to examine the specific cell subtype expressers of scavenger receptors and better inform us of their role within the liver and TMEs.

In addition to directly targeting scavenger receptors for treatment of liver diseases, scavenger receptors may also be useful in the delivery of emerging liver-specific and even cell-specific therapeutics, such as nanoparticles, oligonucleotides, and antibody–drug conjugates. Nanoparticles are small (< 500 nm) quasi-spherical, hollow particles that can be loaded with a range of existing pharmaceutical drugs and utilized to circumvent problems such as solubility and off-target toxicity. 195 Due to the endocytic nature of liver-resident cells, drug-laden nanoparticles passively accumulate within the liver, 195 196 and are known to be internalized by scavenger receptors. 197 Recently, nanoparticle uptake experiments in zebrafish ( *Danio rerio* ) have implicated stabilin-1 and stabilin-2 as key receptors in their clearance by liver endothelial cells and have been utilized to provide proof-of-concept data in the design of targeted and cell-specific drug delivery systems. 198 199 Stabilin-1 and stabilin-2 are also considered to be key molecules in the delivery of oligonucleotide therapeutics. 200 Oligonucleotide therapeutics, such as antisense oligonucleotides, are increasingly being recognized in translational science as an effective therapeutic tool and a number of them have been approved for use in the clinic to treat a range of diseases. 201 Scavenger receptors, such as stabilin-1, stabilin-2, and ASGR1, can be specifically targeted by direct modifications to the oligonucleotides themselves, 200 202 and SR-AI and SCARF1 have been utilized in the delivery of oligonucleotides via viral vectors. 106 Another promising emerging technology in which scavenger receptors have been effectively exploited in preclinical models is antibody–drug conjugation. Anti-CD163 antibody–drug conjugates allow highly efficient delivery of drugs to CD163 ^+^ macrophages, for example, antibody-conjugated dexamethasone displays a high affinity for CD163 and showed a 50-fold more potent anti-inflammatory response than nonconjugated dexamethasone in a rat model of endotoxemia. 203 Furthermore, CD163 antibody-conjugated dexamethasone specifically targeted Kupffer cells and significantly reduced steatohepatitis and fibrosis in a rat model of NAFLD. 204 In addition, this therapeutic strategy may also be valuable in the context of HCC, since CD163 is highly expressed by TAMs, which are indicative of poor patient prognosis. 154 173 Indeed, doxorubicin-containing anti-CD163 liposomes have been shown to reduce tumor growth and enhance monocyte and CD4 ^+^ /CD8 ^+^ T cell infiltration via depletion of immunosuppressive TAMs. 205

Future translational work will hopefully take advantage of the potential of scavenger receptors as biomarkers, to aid in stratifying patients, and as direct therapeutic targets and/or delivery molecules. This could facilitate the design of personalized therapies that target specific scavenger receptors depending on disease stage or cancer risk.

## Conclusions

PRRs play a crucial role in the initial response to foreign pathogens and are a vital link between the innate and adaptive immune response. The literature often highlights the role of TLRs and the inflammasome, but gathering evidence demonstrates that scavenger receptors are also important players in this process. Like the TLR family and inflammasome, they are highly evolutionarily-conserved and recognize a wide array of ligands. The belief that they demonstrate a high level of redundancy is also being brought into question by the use of transgenic murine models, especially in the context of tissue injury.

**Fig. 3:**
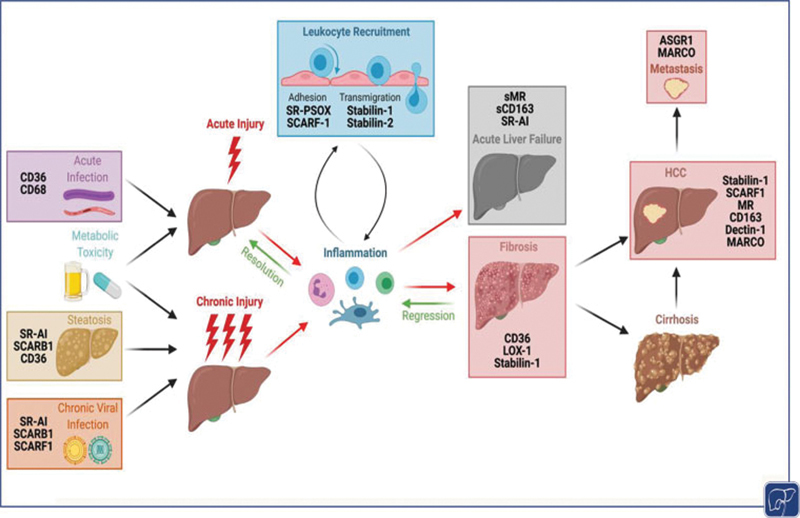
Scavenger receptors have widespread and pleiotropic roles in liver pathophysiology. Acute or chronic liver injury causes inflammation, which if unresolved, can progress to fibrosis or acute liver failure, respectively. Inflammation is underpinned by leukocyte recruitment across the sinusoidal endothelium, and several scavenger receptors have been implicated in adhesion or transmigration steps of the leukocyte adhesion cascade. Although early fibrosis can regress, if noxious stimulus is not removed and liver injury persists, fibrosis will progress to cirrhosis and/or hepatocellular carcinoma (HCC). Figure created using biorender.com.

The liver has a critical role in maintaining homeostasis from a metabolic and immune point of view. Its contribution to tolerance is now well recognized and it can also be the victim of a range of insults which if chronic can lead to a maladaptive wound healing response. The increased expression of scavenger receptors within the hepatic microenvironment contributes to both the metabolic and immune functions of the liver. As discussed, scavenger receptors are a highly diverse superfamily of receptors and, as a result, it is unsurprising that they have been found to play a multitude of roles in the pathogenesis of acute liver injury, chronic liver disease, and HCC ( Fig. 3 ). While these receptors can be expressed on a range of cell types, they are highly expressed on macrophages and endothelial cells. Studies in the context of liver inflammation and fibrosis have demonstrated that scavenger receptor function contributes to leukocyte subset trafficking across endothelium 60 87 and macrophage polarization. 17 79 Given the inflammatory cell infiltration that characterizes all liver diseases and the well-recognized role of macrophages in regulating liver fibrosis, 206 these scavenger receptor properties should impact on the immune and stromal microenvironment of human liver disease and support their potential as therapeutic targets for fibrosis and cancer within the liver.

In this review, we have covered some of the pathways that are regulated by scavenger receptors in the tissue microenvironment of chronic liver injury and carcinogenesis. The potential of scavenger receptors to positively influence the liver microenvironment in the disease setting and their potential to act as therapeutic targets are only just starting to be realized, with a small number of early-phase clinical trials having been undertaken to date. However, we predict that this unique family of receptors, which often have a cell-specific expression, could be highly attractive in regulating distinct inflammatory pathways and in targeting specific sites of disease.

## Main Concepts and Learning Points

- Inflammation is a key pathological component of acute and chronic liver injuries, cirrhosis, and hepatocellular carcinoma (HCC); however, the molecules and pathways involved in hepatic inflammation are yet to be fully elucidated.
- Scavenger receptors are a highly diverse superfamily of receptors which play an important role in homeostasis, but are also associated with the pathophysiology of several inflammatory diseases.
- Scavenger receptors are highly expressed within the liver, particularly in hepatic macrophages and liver sinusoidal endothelial cells (LSECs), and regulate discrete immune pathways in response to hepatic infection and sterile injury.
- Despite sharing the recognition of several ligands, scavenger receptors have been shown to play nonredundant roles in shaping the stromal response in preclinical models of liver injury.
- Developing agents that target scavenger receptors could be a promising approach in treating fibrosis and carcinogenesis within the hepatic microenvironment.
