# Erratum to: Scavenger Receptors: Novel Roles in the Pathogenesis of Liver Inflammation and Cancer

**DOI:** 10.1055/s-0041-1739050

**Published:** 2021-11-18

**Authors:** Daniel A. Patten, Alex L. Wilkinson, Ayla O'Keeffe, Shishir Shetty

**Affiliations:** 1 National Institute for Health Research Birmingham Liver Biomedical Research Unit, Institute of Immunology and Immunotherapy, University of Birmingham, Birmingham, United Kingdom; 2 Centre for Liver and Gastrointestinal Research, Institute of Immunology and Immunotherapy, University of Birmingham, Birmingham, United Kingdom

## Erratum

The authors have informed the Publisher that a conflict of interest was missed in the above-mentioned article published online on September 22, 2021. DOI of the article is 10.1055/s-0041-1733876 . The declaration of the conflict of interest is as follows:

Dr. Shishir Shetty declares that he received consultation fees from Faron Pharmaceuticals, and the payment was managed by his institution, University of Brimingham, Birmingham, United Kingdom.

